# The Impact of Respiratory Infection of H9N2 Avian Influenza Virus on Intestinal Microbiota in Chickens via Gut-Lung Axis

**DOI:** 10.3390/microorganisms14102219

**Published:** 2026-10-02

**Authors:** Yixiang Wang, Yuan Fang, Kehui Zhang, Xuanrong Wang, Guanlong Xu, Jie Wang, Zhaofei Wang, Yuqiang Cheng, Heng’an Wang, Yaxian Yan, Jianhe Sun, Jingjiao Ma

**Affiliations:** 1 Shanghai Key Laboratory of Veterinary Biotechnology, Key Laboratory of Urban Agriculture (South), Ministry of Agriculture, School of Agriculture and Biology, Shanghai Jiao Tong University, Shanghai 200240, China; 2 Department of General Surgery, Peking Union Medical College Hospital, Chinese Academy of Medical Sciences and Peking Union Medical College, Beijing 100730, China; 3 State Key Laboratory of Complex, Severe, and Rare Diseases, Peking Union Medical College Hospital, Chinese Academy of Medical Sciences and Peking Union Medical College, Beijing 102602, China; 4 China Institute of Veterinary Drug Control, Beijing 100081, China

**Keywords:** chicken, low pathogenic avian influenza (LPAI), H9N2 subtype, lung-gut axis, microbiota

## Abstract

In the poultry industry, infection with low-pathogenicity avian influenza viruses is often accompanied by atypical clinical manifestations, such as diarrhea, the underlying mechanisms of which are yet to be elucidated. In this study, ten chickens were randomly divided into two groups (*n* = 5 per group). One group is infected with the H9N2 subtype of avian influenza virus to simulate the clinical infection. Bronchioalveolar lavage fluid (BALF), as well as cecal and colonic contents samples, was collected at 5 days post inoculation (dpi), and 16S rRNA sequencing was employed to analyze the alterations of the pulmonary and intestinal microbiota. Our results showed that H9N2 infection did not significantly alter the alpha or beta diversity of either the gut or lung microbiota. LEfSe analysis revealed that the infected group exhibited enrichment of *Oscillospira* and *Fournierella* in the gut and *Delftia* and *Pelomonas* in the lung, whereas the control group showed enrichment of *Bacteroides*, *Enorma*, *Anaerostignum*, and *Collinsella* in the gut and *Mycoplasma* in the lung. Procrustes analysis showed no significant overall structural association between the two niches, while Spearman correlation analysis identified 54 significant cross-site microbial correlations. BugBase analysis indicated that the lung microbiota in the infected group exhibited significantly enhanced phenotypes related to potential pathogenicity, Gram-negative status, and stress tolerance. Collectively, these findings suggest that H9N2 may reshape the composition, function, and cross-site interaction networks of specific microbial taxa. Viral infection may be associated with cross-site alterations in the gut and lung microbiota via the “gut-lung axis”, with gut microbiota perturbations potentially influencing pulmonary susceptibility to secondary infections. This highlights the gut microbiota as a potential target for modulating respiratory pathogen colonization and reducing antibiotic use, while providing a novel theoretical basis for understanding the gut-lung axis in avian influenza infection in poultry.

## 1. Introduction

H9N2 subtype avian influenza virus primarily infects the respiratory tract of chickens. Although classified as low-pathogenic, it can induce immunosuppression, predisposing infected birds to secondary bacterial infections [1,2,3], and has occasionally been reported to cause gastrointestinal symptoms such as diarrhea, further complicating the clinical presentation [4]. How does an infection that is primarily localized in the upper respiratory tract affect the distal intestinal environment? And what underlies its propensity to predispose the host to secondary bacterial infections? Despite the clinical significance of these questions, the impact of H9N2 infection on the respiratory microbiota of chickens remains poorly understood, particularly regarding its potential mediation through the gut-lung axis. Elucidating the regulatory mechanisms of the gut-lung axis may provide new insights into improving poultry health through modulation of the respiratory microbiota.

The lung, long considered sterile, is now known to harbor a dynamic microbial community that plays a significant role in host immune regulation [5,6]. The gut microbiota constitutes a highly complex and taxonomically diverse microbial ecosystem and contributes to nutrient metabolism, immune modulation, and maintenance of overall health. The bidirectional communication between the lungs and the intestines has been termed the “gut-lung axis”, which represents a functional cross-talk network between these two organs. In poultry, research has demonstrated that the gut microbiota plays a critical role in modulating immune responses at distant sites, including the respiratory tract [7]. Studies using germ-free and conventional chickens have provided direct evidence that the gut-lung axis is functional in chickens. The importance of early-life gut colonization in chickens has been emphasized, as it influences immune system development and may impact respiratory health through the gut-lung axis [7,8]. Furthermore, experimental evidence has shown that gut microbiota-derived metabolites, particularly short-chain fatty acids (SCFAs) such as butyrate, can exert antiviral functions in chicken respiratory epithelial cells by modulating interferon-stimulated genes [8]. These findings indicate that the gut-lung axis is established early in life and may play a critical role in coordinating host immune development and defense against respiratory pathogens in poultry. Multiple studies have demonstrated that respiratory influenza virus infection affects the gut microbial community across subtypes, including H1N1, H3N2, H5N1, and H7N9 [9]. Conversely, differences in the gut microbial composition can influence the prognosis of these viral infections, highlighting a bidirectional gut-lung interplay. In chickens, H9N2 infection has been shown to significantly alter the upper respiratory microbiota, with Lactobacillaceae highly enriched during the acute phase [10]. Meanwhile, the gut microbiota plays a critical role in modulating host immune responses to respiratory pathogens. A recent review further consolidated these findings, linking avian influenza virus infection with gut dysbiosis and altered immune status in chickens, noting that H9N2 infection promotes depletion of beneficial genera such as *Lactobacillus* and *Enterococcus* while enriching Proteobacteria-associated pathogens [11]. Beyond viral infections, a microbial consortium constructed from gut microbes of Chinese native chicken breeds has been shown to protect chicks against *Salmonella* infection, further supporting the role of the gut microbiota in defending against respiratory and systemic pathogens in poultry [12].

While these studies have established that H9N2 infection can independently alter the upper respiratory microbiota [10] and the intestinal microbiota [13], whether and how these two compartments interact at the microbial level during infection remains unexplored. Therefore, this study was designed to investigate how H9N2 infection reshapes the gut and lung microbial communities in chickens and to explore whether pulmonary infection drives gut microbial dysbiosis via the gut-lung axis and thereby alters host susceptibility to secondary bacterial infections. By integrating compositional and functional characterization of both niches, we aim to describe the microbial changes associated with H9N2 infection along the gut-lung axis, ultimately informing rational microbiota-targeted measures for improving poultry health.

## 2. Materials and Methods

### 2.1. Viruses

An avian influenza virus strain A/Avian/SH01/2018 (H9N2) was obtained from the CDC of Shanghai, China, and maintained in the laboratory. The virus was amplified in embryos before use. The virus titers were examined by the Hemagglutination (HA) assay. Briefly, serially dilute virus in PBS in a 96-well plate. Add an equal volume of red blood cells (e.g., 0.5–1% suspension). Incubate at room temperature for 30–60 min, then read results. The highest dilution giving a positive (no button) is the HA titer. The allenoic fluid was then tested for 50% Egg Infectious Dose (EID_50_). Briefly, serially dilute the virus in a suitable medium. Inoculate each dilution into multiple embryonated chicken embryos at 37 °C for 72 h. Check for viral presence using the HA assay in harvested allantoic fluid. Calculate the EID_50_/mL using the Reed & Muench method [14].

### 2.2. Animal Study

Ten 4-week-old SPF white Leghorn chickens were purchased from Jiangsu Boehringer Ingelheim Weitong Biotechnology Co., Ltd. (Nantong, China) with both male and female. Chickens were housed in isolators with controlled temperature (33 °C) and humidity (45–55%). No antibiotics, probiotics, or vaccines were administered to the birds. The compound feed for SPF layer chickens during the brooding period was purchased from Keao Xieli Feed Co., Ltd. (Tianjin, China) (Ingredients: corn, soybean meal, extruded soybean, dicalcium phosphate, limestone powder, choline, multivitamins and minerals, etc.). Ten chickens were randomly (arbitrarily selected) separated into two groups with 5 chickens in each group. One group is for virus inoculation, and another is for MOCK control. Chickens within the same treatment group were housed collectively in the same isolator. Inoculate the chicken intranasally and intraocularly with 10^5^ EID_50_ viruses in 50 μL PBS, then observe the chickens for five days. The MOCK control group received 50 μL PBS through the same routes. At 5 dpi, all chickens were euthanasia and necropsied. Since previous studies have reported that H9N2-induced microbiota alterations are most pronounced during the acute phase, particularly around days 2–5 post-infection [15]. Lung lavage fluid was obtained by washing the chicken lung with sterile PBS. Approximately 1 g of cecal and colonic contents was individually collected from each chicken. Samples were kept at −80 °C.

### 2.3. 16S rRNA Sequencing and Analysis

Samples of lung lavage fluid and intestinal contents were collected for 16S rRNA sequencing analysis. All specimens were aseptically placed into sterile plastic bags, immediately transferred to the laboratory on dry ice, and stored at −80 °C for DNA high-throughput sequencing. Total microbial genomic DNA was extracted from the samples using the E.Z.N.A.^®^ Stool DNA Kit (Omega Bio-tek, Norcross, GA, USA). The hypervariable V3-V4 region of the bacterial 16S rRNA gene was amplified using the primer pairs 338F (5′-ACTCCTACGGGAGGCAGCAG-3′) and 806R (5′-GGACTACHVGGGTWTCTAAT-3′) [16]. Purified amplicons were pooled in equimolar amounts and subjected to paired-end sequencing on an Illumina NextSeq 2000 platform (Illumina, San Diego, USA) following the standard protocols provided by Majorbio Bio-Pharm Technology Co., Ltd. (Shanghai, China). The raw sequencing data have been deposited in the NCBI Sequence Read Archive (SRA) under accession number PRJNA1531431. Bioinformatic analysis of the gut microbiota was carried out using the Majorbio Cloud platform (https://cloud.majorbio.com accessed on 5 January 2026).

Alpha diversity was evaluated using Chao1, Shannon, and Simpson indices. Beta diversity was assessed by PERMANOVA (999 permutations) based on Bray-Curtis distance and visualized by PCoA. Differentially abundant taxa between groups were identified using LEfSe (LDA > 2, *p* < 0.05) as appropriate. Functional potential was predicted using PICRUSt2, and phenotypic traits were predicted using BugBase. All pairwise comparisons were performed using a two-sided Wilcoxon rank-sum test (Mann-Whitney U-test). All statistical analyses were performed using the Majorbio Cloud platform (https://cloud.majorbio.com accessed on 29 May 2026).

## 3. Results

### 3.1. Overall Microbiome Diversity Remained Unchanged After Infection

In our study, clinical signs were monitored daily after infection. Chickens exhibited depression and conjunctivitis, which are consistent with H9N2 infection via the intranasal and intraocular routes. These signs appeared around 3 days post-infection and persisted through day 5. Mild diarrhea was occasionally observed in some infected birds. No obvious clinical signs were observed in the control group throughout the study period.

To assess the impact of H9N2 infection on the microbial communities in chickens, we compared the alpha and beta diversity of both intestinal and lung microbiota between the infected and control groups. Alpha diversity was evaluated using the Chao1 richness estimator, as well as the Shannon and Simpson diversity indices. Beta diversity was assessed by principal coordinate analysis (PCoA) based on Bray-Curtis distances.

**Figure 1:**
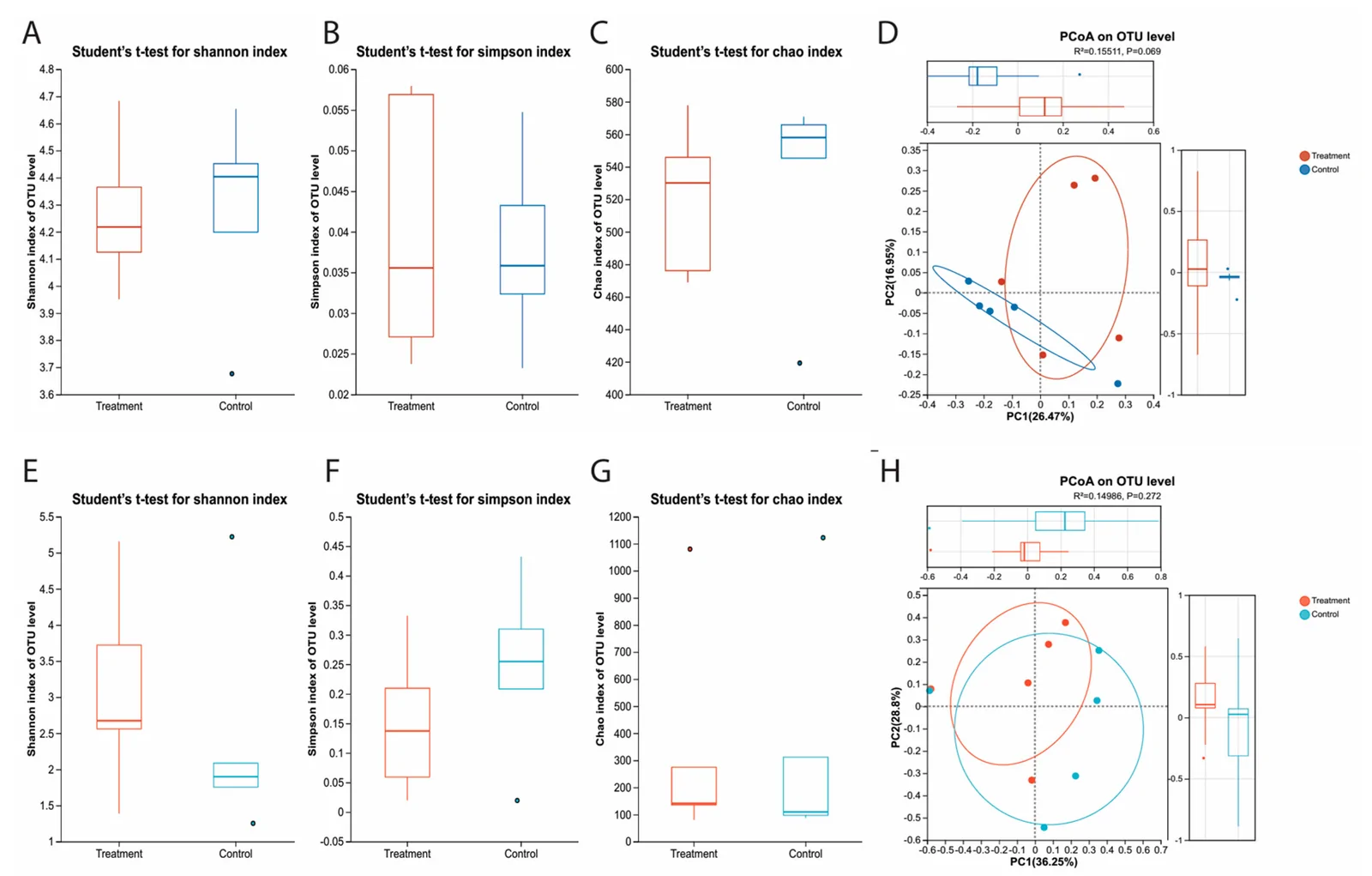
Comparison of gut and lung microbiota diversity after H9N2 infection. (**A**–**C**) Alpha-diversity of gut microbiome between treatment and control groups based on Chao1, Shannon, and Simpson indices. The box represented the interquartile range (IQR) between the first and the third quartiles, and the midline represented the median. (**D**) Principal coordinates analysis (PCoA) for gut microbiome between treatment and control groups based on bray-curtis distance. (**E**–**G**) Alpha-diversity of lung microbiome between treatment and control groups. (**H**) PCoA for lung microbiome between treatment and control groups based on bray-curtis distance.

For the intestinal microbiota, no significant differences were observed in alpha diversity between the infected and control groups, as measured by the Chao1, Shannon, and Simpson indices (*p* > 0.05) (Figure 1A–C). Consistently, beta diversity analysis based on PCoA of Bray-Curtis distances revealed no distinct clustering of intestinal microbial communities between the two groups (PERMANOVA, *p* > 0.05), indicating that no significant change in the overall structure of the intestinal microbiota was detected upon viral infection (Figure 1D).

Similarly, analysis of the lung microbiota revealed no significant differences in alpha diversity between the infected and control groups (Chao1, Shannon, and Simpson indices, all *p* > 0.05) (Figure 1E–G). Beta diversity analysis, as visualized by PCoA, also failed to separate the lung microbial communities of infected chickens from those of the controls (PERMANOVA, *p* > 0.05) (Figure 1H), suggesting no significant alteration in the overall composition of the lung microbiota was detected following H9N2 infection. These results indicate that under the current experimental conditions, H9N2 infection did not induce significant shifts in either the diversity or overall community structure of the intestinal and lung microbiota in chickens.

### 3.2. Gut and Lung Microbiota Exhibit Distinct Composition Profiles

**Figure 2:**
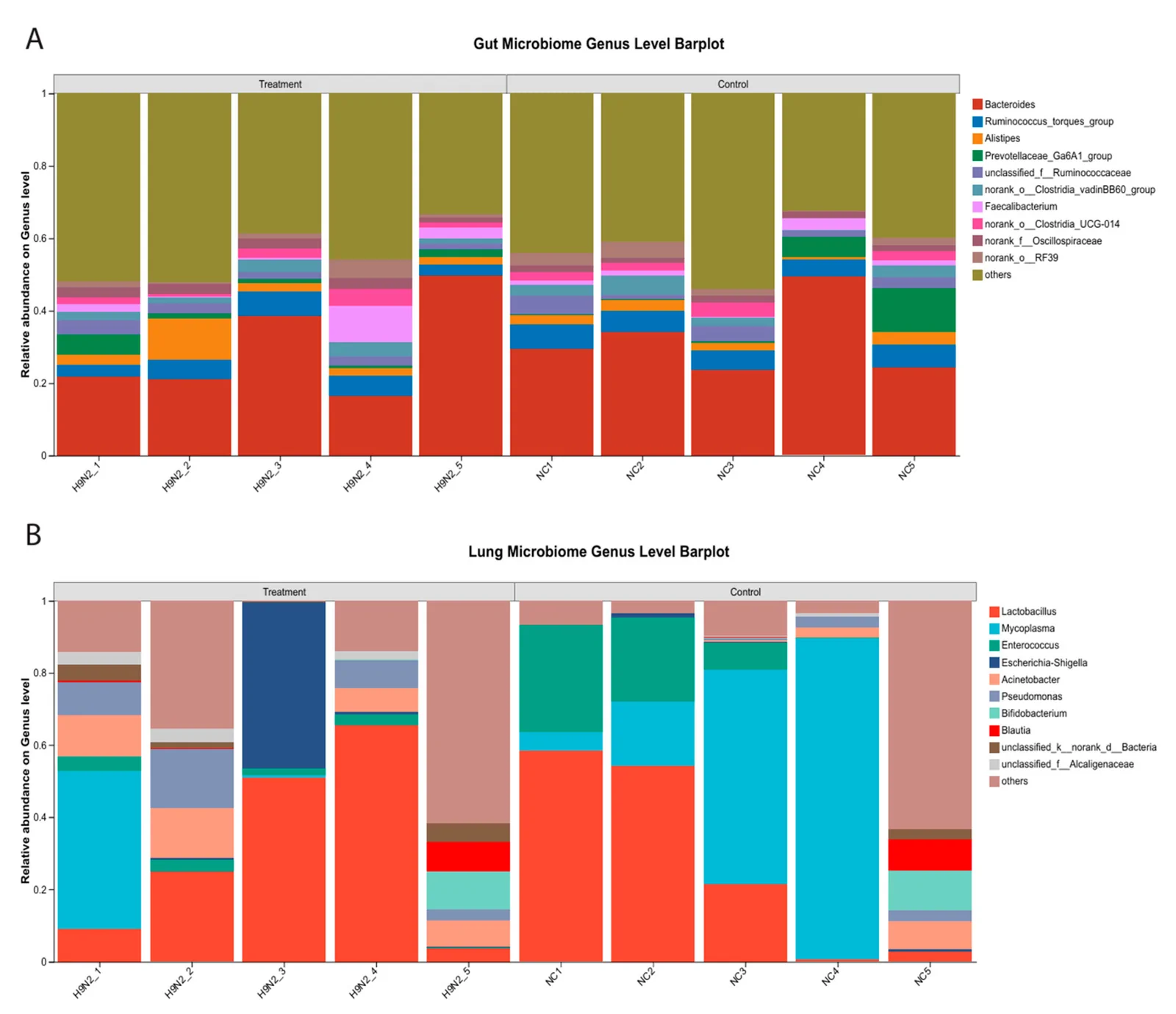
Relative abundance of top 10 bacterial genera in the gut and lung microbiota. Stacked bar chart showing the relative abundance of the top 10 genera in the intestinal microbiota (**A**) and lung microbiome (**B**) of H9N2-infected and control chickens. Taxa with relative abundance below the top 10 were pooled as “Others”.

To characterize the overall structure of the microbial communities, we analyzed the relative abundance of bacterial taxa at the genus level in both intestinal and lung samples from infected and control chickens. At the genus level, the intestinal microbiota of chickens was predominantly composed of several core taxa (Figure 2A). In descending order of abundance, the top 10 genera were *Bacteroides*, *Ruminococcus torques group*, *Alistipes*, *Prevotellaceae* Ga6A1 group, unclassified Ruminococcaceae, unclassified *Clostridia* vadinBB60, *Faecalibacterium*, unclassified *Clostridia* UCG-014, unclassified Oscillospiraceae, and unclassified *RF39*. A notable proportion of sequences were classified as Others, representing genera with lower abundance. The microbial composition of lung samples differed markedly from that of the intestinal samples (Figure 2B). In descending order of relative abundance, the top 10 taxa were *Lactobacillus*, *Mycoplasma*, *Enterococcus*, *Escherichia-Shigella*, *Acinetobacter*, *Pseudomonas*, *Bifidobacterium*, *Blautia*, unclassified Bacteria, and unclassified Alcaligenaceae. Collectively, these results reveal distinct microbial composition profiles between the intestinal and lung niches in chickens. The intestinal microbiota was characterized by a diverse array of genera commonly associated with gut health, including *Bacteroides*, *Alistipes*, and *Faecalibacterium*. In contrast, the lung microbiota was dominated by *Lactobacillus*, *Mycoplasma*, and *Enterococcus*, with a notable presence of potential opportunistic pathogens, reflecting the distinct ecological niches of these anatomical sites.

### 3.3. Differential Microbial Taxa in Gut-Lung Axis

**Figure 3:**
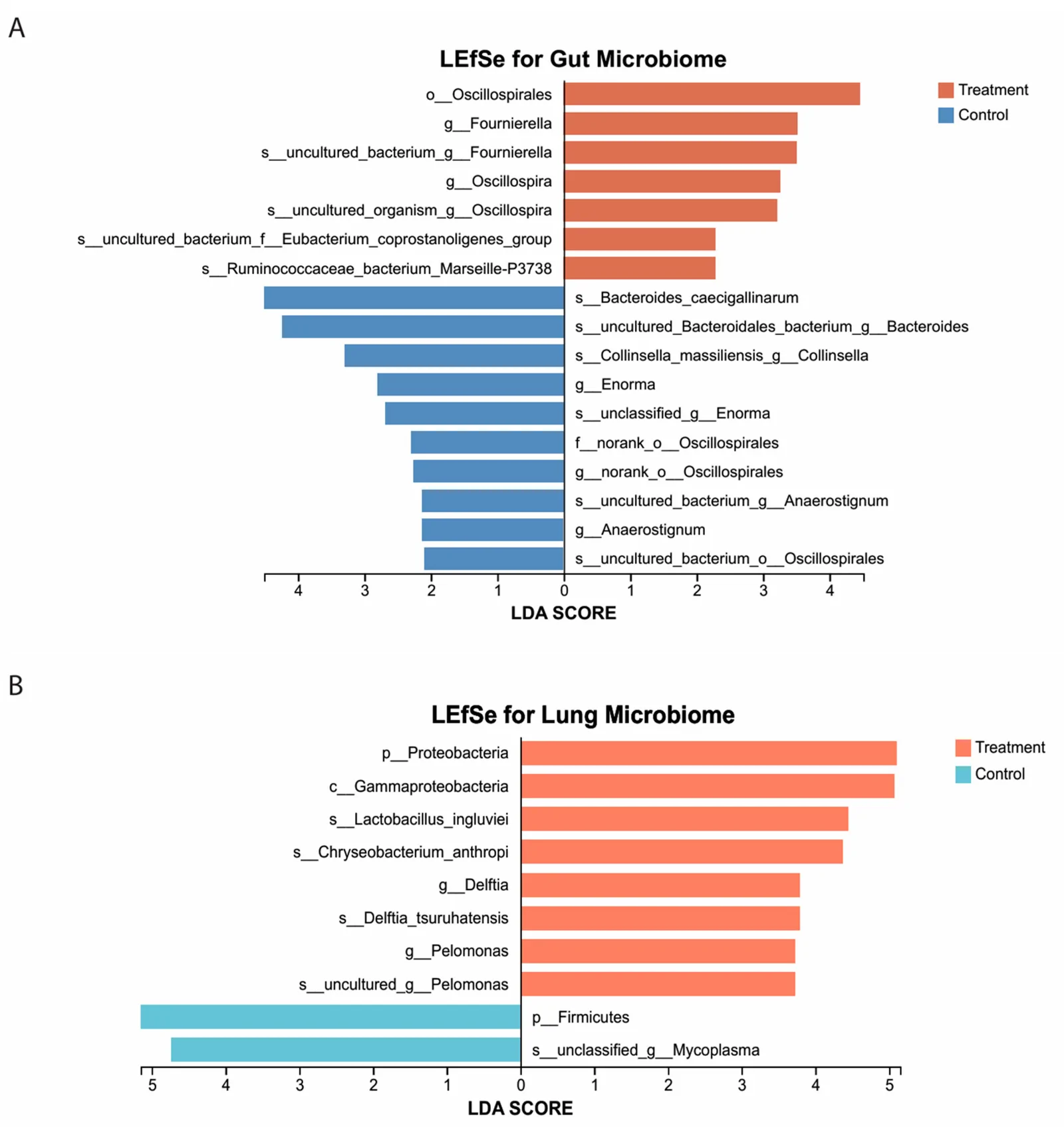
Differential taxa in H9N2-infected group and control. Altered gut (**A**) and lung (**B**) taxa identified by LEfSe analysis (LDA > 2).

To identify specific bacterial taxa associated with H9N2 infection, linear discriminant analysis effect size (LEfSe) was performed on both intestinal and lung microbiota data, using an LDA score > 2.0 and *p* < 0.05 as thresholds. For the intestinal microbiota, a total of 17 differential taxa were identified between the treatment and control groups (Figure 3A). In the treatment group, 7 enriched taxa were predominantly affiliated with the order Oscillospirales. Families Ruminococcaceae and Oscillospiraceae showed higher abundance following H9N2 infection, including the species *Ruminococcaceae bacterium* Marseille-P3738 and an uncultured organism from the genus *Oscillospira*. Additionally, the family Eubacterium coprostanoligenes group and the genus *Fournierella* were also enriched in the treatment group. In the control group, 10 enriched taxa were identified spanning multiple phyla. The phyla Bacteroidota and Actinobacteriota were enriched in control animals. For Bacteroidota, the genus *Bacteroides* was enriched, including the species *Bacteroides caecigallinarum* and uncultured *Bacteroidales bacterium*. From Actinobacteriota, the species *Collinsella massiliensis* and the genus *Enorma* showed higher abundance in controls. In Firmicutes, the genus *Anaerostignum* was enriched. Additionally, multiple taxa classified as norank Oscillospirales were also enriched in the control group, indicating a compositional shift within the Oscillospirales between the two groups.

A total of 10 differential taxa were identified between the treatment and control groups in the lung microbiota (Figure 3B). In the treatment group, higher taxonomic levels were enriched, including the phylum Proteobacteria, the class Gammaproteobacteria, and the order *Burkholderiales*. At the family level, Comamonadaceae was enriched. Within this family, the genus *Delftia*, including the species *Delftia tsuruhatensis*, and the genus *Pelomonas* showed significantly higher abundance following infection. Beyond *Proteobacteria*, the species *Chryseobacterium anthropi* and *Lactobacillus ingluviei* were also more abundant in H9N2-infected chickens. In the control group, only the phylum *Firmicutes* and the genus *Mycoplasma* were enriched.

### 3.4. Associations Between Intestinal and Lung Microbiota

**Figure 4:**
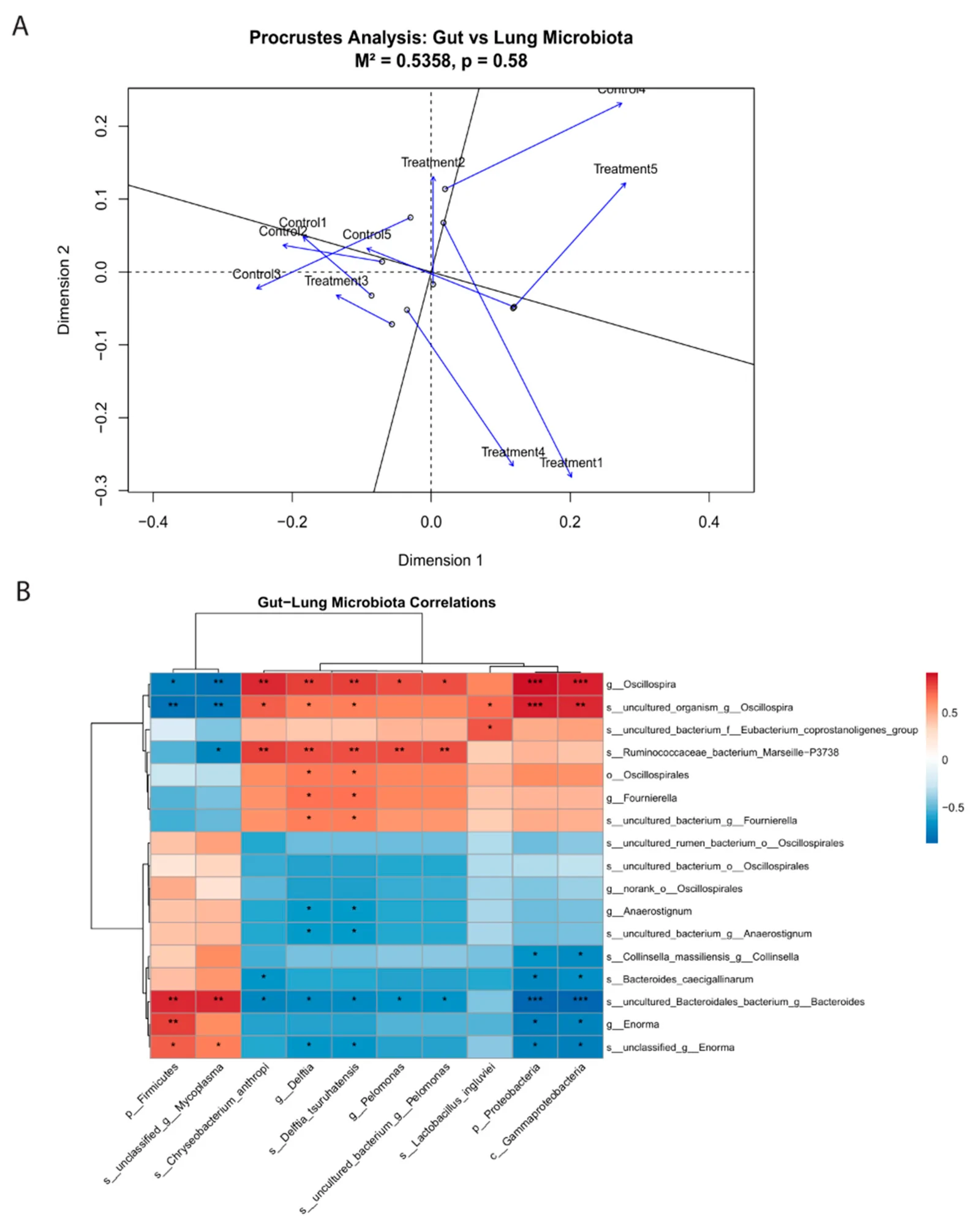
Cross-site microbial correlations between gut and lung microbiota. (**A**) Procrustes analysis of the gut and lung microbiota. The gut microbiota was ordinated by PCoA based on Bray–Curtis distances, and the lung ordination was superimposed by Procrustes rotation. Each point represents an individual sample. Arrows connect the corresponding gut and lung samples, with arrow length indicating the magnitude of residual mismatch between the two microbiotas; shorter arrows indicate greater congruence between the gut and lung community structures. No significant overall structural association was detected (M^2^ = 0.5358, *p* = 0.580). (**B**) Spearman correlation heatmap showing associations between differentially abundant intestinal taxa and differentially abundant lung taxa. Cells represent Spearman’s rank correlation coefficients (r), with red indicating positive correlations and blue indicating negative correlations: * *p* < 0.05, ** *p* < 0.01, *** *p* < 0.001. All significant correlations had |r| > 0.6.

To investigate the potential interplay between intestinal and lung microbial communities along the gut-lung axis, Procrustes analysis was performed to assess the overall structural similarity between the two niches (Figure 4A). No significant correlation was detected between the global community structures of gut and lung microbiota (M^2^= 0.5358, *p* = 0.580), suggesting no significant global association between the overall microbial compositions of these two anatomical sites. Given the absence of a significant global structural association, Spearman correlation analysis was performed to explore potential relationships between specific differentially abundant bacterial taxa in the gut and lung, identified by LEfSe (LDA > 2.0, *p* < 0.05). As shown in Figure 4B, a total of 54 significant correlations were identified between intestinal and lung taxa.

The phylum Firmicutes in the lung showed positive correlations with multiple intestinal taxa, including the genus *Enorma*, the species unclassified *Enorma*, and the uncultured *Bacteroidales bacterium* from the genus *Bacteroides*. Conversely, Firmicutes exhibited negative correlations with the genus *Oscillospira* and the uncultured organism from the genus *Oscillospira*. The phylum *Proteobacteria* and its subordinate class Gammaproteobacteria in the lung displayed similar correlation patterns. Both were positively correlated with the genus *Oscillospira* and the species uncultured organism from the genus *Oscillospira*, and negatively correlated with the genus *Enorma*, the species unclassified *Enorma*, *Collinsella massiliensis*, *Bacteroides caecigallinarum*, and uncultured *Bacteroidales bacterium*.

At the genus level, lung *Delftia* and its species *Delftia tsuruhatensis* showed parallel correlation patterns. Both were positively correlated with the order Oscillospirales, the genus *Oscillospira*, the genus *Fournierella*, the uncultured organism from the genus *Oscillospira*, the uncultured bacterium from the genus *Fournierella*, and *the Ruminococcaceae bacterium Marseille-P3738*. Negative correlations were observed with the genus *Anaerostignum*, the species unclassified *Enorma*, the uncultured *Bacteroidales bacterium*, and the uncultured *bacterium* from the genus *Anaerostignum*. Lung *Pelomonas* and its uncultured species showed positive correlations with the genus *Oscillospira* and *Ruminococcaceae bacterium* Marseille-P3738 and a negative correlation with the uncultured *Bacteroidales bacterium*. Lung *Chryseobacterium anthropi* was positively correlated with the genus *Oscillospira*, the species uncultured organism from the genus *Oscillospira*, and *the Ruminococcaceae bacterium* Marseille-P3738, and negatively correlated with *Bacteroides caecigallinarum* and the uncultured *Bacteroidales bacterium*. Lung *Lactobacillus ingluviei* showed positive correlations with uncultured *bacterium* from the *Eubacterium coprostanoligenes* group and the species of uncultured organisms from the genus *Oscillospira*. Finally, lung *Mycoplasma* was positively correlated with the species unclassified *Enorma* and uncultured *Bacteroidales bacterium* and negatively correlated with the genus *Oscillospira*, the species uncultured organism from the genus *Oscillospira*, and *Ruminococcaceae bacterium Marseille-P3738*.

### 3.5. Functional and Phenotypic Prediction of Gut and Lung Microbiota

To predict the functional potential of gut and lung microbiota, PICRUSt2 analysis was performed on 16S rRNA sequencing data. KEGG pathway differences between treatment and control groups were analyzed for each niche.

**Figure 5:**
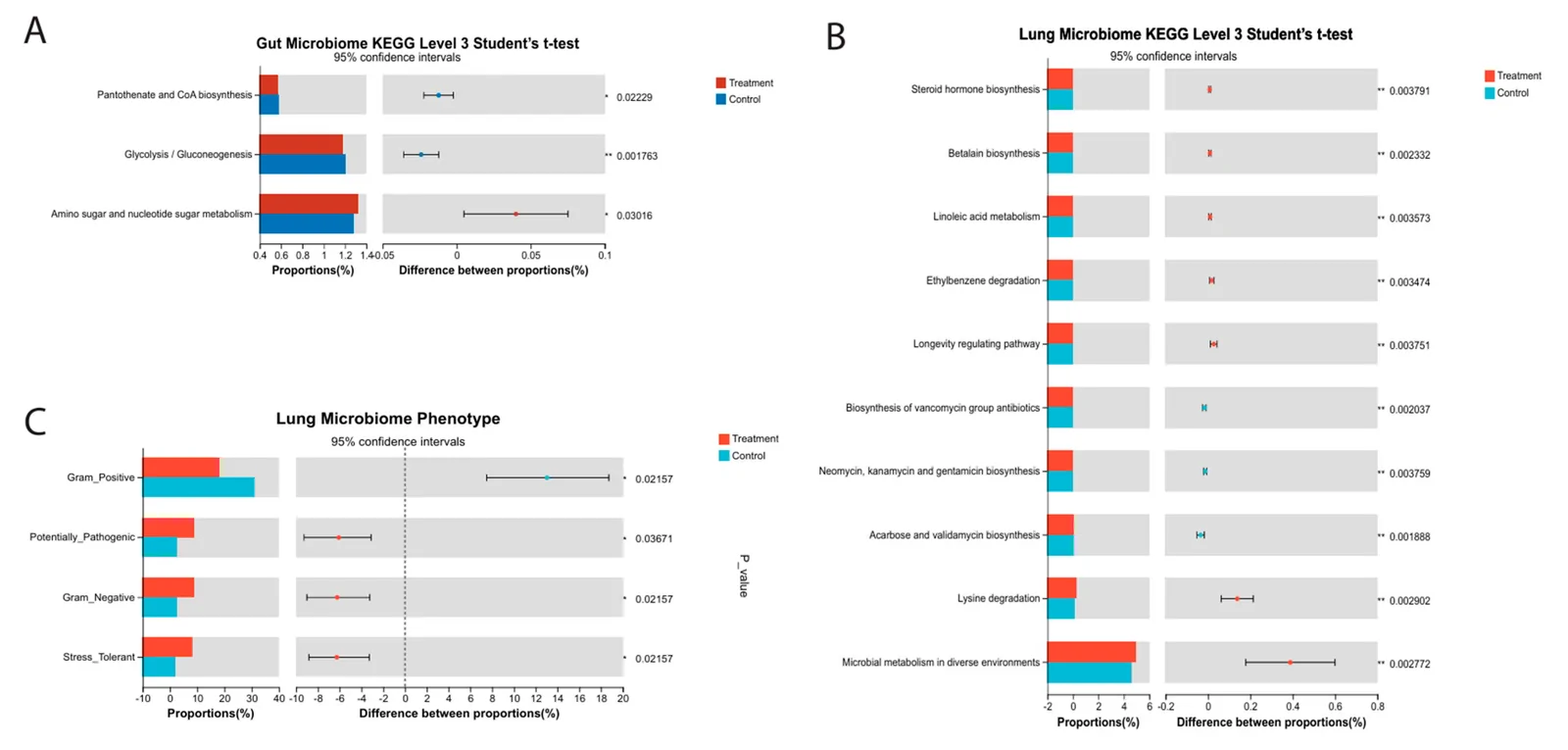
Functional alterations of gut and lung microbiota (**A**) KEGG pathways significantly altered in the intestinal microbiota between H9N2-infected and control groups. (**B**) Top 10 KEGG pathways in the lung microbiota. (**C**) BugBase-predicted phenotypic traits of the lung microbiota. * *p* < 0.05, ** *p* < 0.01.

For the gut microbiota, three KEGG pathways showed significant differences between groups. Pantothenate and CoA biosynthesis and Glycolysis/Gluconeogenesis were enriched in the control group, while Amino sugar and nucleotide sugar metabolism was enriched in the treatment group (Figure 5A). For the lung microbiota, a total of 86 KEGG pathways showed significant differences between groups. The top 10 pathways with the smallest *p*-values were presented in Figure 5B, and the complete list was provided in Table S1**.** Among these, three pathways were enriched in the control group, including Acarbose and validamycin biosynthesis, Biosynthesis of vancomycin group antibiotics, and Neomycin, kanamycin and gentamicin biosynthesis. The remaining seven pathways were enriched in the treatment group, including Betalain biosynthesis, Microbial metabolism in diverse environments, Lysine degradation, Ethylbenzene degradation, Linoleic acid metabolism, Longevity regulating pathway, and Steroid hormone biosynthesis.

BugBase analysis was performed to predict phenotypic traits of the gut and lung microbiota. No significant differences in phenotypic traits were observed in the gut microbiome between the treatment and control groups. For the lung microbiota, several phenotypic traits differed significantly between groups. The treatment group showed increased abundance of Potentially Pathogenic, Gram Negative, and Stress Tolerant phenotypes compared to the control group, while Gram Positive phenotype was decreased in the treatment group (Figure 5C).

## 4. Discussion

In this study, we found that H9N2 infection did not significantly alter the alpha or beta diversity of either the intestinal or lung microbiota. This observation differs from some previous reports, which have shown that H9N2 infection can reduce both respiratory and intestinal microbial diversity in chickens, accompanied by depletion of beneficial genera and enrichment of Proteobacteria [10,15,17,18]. The discrepancies between our study and those reports may arise from differences in experimental conditions, including chicken strain, age, housing environment, infection dose, and sampling time points. Chrzastek et al. [15] showed that H9N2 reduces colon alpha diversity specifically during the acute phase (days 2–3), with partial recovery thereafter. Our sampling occurred outside this window. Viral dose and strain may also affect the disruption, with our milder infection being insufficient to alter overall diversity. Our results revealed that the intestinal microbiota of chickens was predominantly composed of genera associated with gut health, such as *Bacteroides*, *Alistipes*, and *Faecalibacterium*. In contrast, the lung microbiota was dominated by *Acinetobacter*, *Pseudomonas*, and *Mycoplasma*, with a notable presence of potentially opportunistic pathogens. These findings demonstrate distinct compositional profiles between the intestinal and respiratory microbiota in chickens, reflecting their different niche characteristics.

In this study, LEfSe analysis revealed that H9N2 infection induced distinct enrichment patterns in the intestinal and lung microbiota. In the intestinal microbiota, the infected group was enriched with Oscillospirales, *Ruminococcaceae*, and the *Eubacterium coprostanoligenes* group, taxa involved in fiber degradation and cholesterol metabolism [19,20]. Recent research has confirmed that H9N2 infection can induce dose-dependent alterations in the gut microbiota, particularly the depletion of *Prevotella* species [21]. The control group was enriched with the phyla Bacteroidota and Actinobacteriota, suggesting that H9N2 infection may lead to a relative reduction in these beneficial microbial communities. It should be noted that Oscillospirales and *Ruminococcaceae* are SCFA-producing taxa. However, these findings are derived from PICRUSt2 prediction, which estimates the potential metabolic capacity of the microbiota rather than actual metabolite production. While previous studies have provided direct evidence that butyrate can regulate interferon-stimulated genes in chicken respiratory epithelial cells [8], whether these pathways are functionally engaged in our experimental system remains to be determined. Future studies incorporating targeted metabolomic profiling of SCFAs and bile acids, combined with cytokine assays, will be essential to establish a causal link between gut microbial metabolic activity and pulmonary immune modulation during H9N2 infection. In the lung microbiota, Proteobacteria was enriched in the H9N2-infected group, a phylum that commonly expands in respiratory infections, and its increase is often regarded as a marker of microbial dysbiosis [22], including the genera *Delftia* and *Pelomonas*, which are ubiquitous in environmental niches but recognized as opportunistic pathogens. Notably, the pulmonary enrichment of *Lactobacillus ingluviei*, a species typically resident in the avian digestive tract, suggests that the pulmonary microenvironment may selectively favor this taxon under the conditions studied. In contrast, the control group was enriched with *Firmicutes* and *Mycoplasma*. *Mycoplasma*, a known poultry pathogen, was enriched in the control group, but the biological significance of this enrichment remains to be determined. Collectively, the differential enrichment patterns observed between the intestinal and pulmonary microbiota indicate that H9N2 infection elicits distinct microbial response strategies in the two ecological niches. In the gut, the response primarily involves metabolic modulation, whereas in the lung, it favors the expansion of opportunistic pathogens, likely driven by local microenvironmental changes such as inflammation and increased mucus secretion.

Procrustes analysis failed to detect a significant correlation between the overall structures of the intestinal and pulmonary microbiota. Nevertheless, the presence of locally significant cross-organ correlations suggests a potential association between the gut and the lung microbiota. In this study, we found that pulmonary Proteobacteria and Gammaproteobacteria were positively correlated with the intestinal genus *Oscillospira*, while negatively correlated with intestinal *Enorma*, *Collinsella*, and *Bacteroides caecigallinarum*. Proteobacteria, particularly the Gammaproteobacteria class, are commonly regarded as a marker of microbial dysbiosis in respiratory infections [9], and previous studies have reported that H9N2 infection leads to an increase in Proteobacteria and *Escherichia-Shigella* in chickens [13,23]. The expansion of pulmonary Proteobacteria was associated with an increase in intestinal *Oscillospira* and a decrease in Bacteroides, possibly reflecting the differential impact of systemic inflammatory responses on the two niches. *Mycoplasma* was enriched in the lungs of the control group, showing a positive correlation with intestinal *Enorma* and a negative correlation with *Oscillospira*. Its relative reduction in the infected group may reflect the suppressive effect of an altered respiratory microenvironment on the original commensal microbiota, and this change is statistically correlated with fluctuations in specific intestinal taxa. Pulmonary *Delftia* and *Pelomonas* were also positively correlated with intestinal *Oscillospira* and *Ruminococcaceae*. Recent studies have demonstrated that H9N2 infection induces dose-dependent alterations in the gut microbiota, particularly the depletion of *Prevotella,* while probiotic intervention can ameliorate infection by remodeling the gut microbial community [21], suggesting that specific gut taxa, such as *Oscillospira*, may play a functional role in modulating pulmonary infection outcomes. These findings reveal statistically significant associations between the gut and lung microbiota, while their biological significance remains to be explored.

Based on PICRUSt2-based functional prediction, H9N2 infection was predicted to exert differential effects on the intestinal and lung microbiota. Only three KEGG pathways were altered in the gut, whereas 86 pathways were changed in the lung. In the intestinal microbiota, the control group was enriched in pantothenate and CoA biosynthesis and glycolysis/gluconeogenesis pathways, while the infected group was enriched only for the amino sugar and nucleotide sugar metabolism pathway, a pattern that may suggest enhanced proliferation of Gram-negative bacteria and biofilm formation under inflammatory conditions. The functional alterations in the lung microbiota were more complex than those in the gut microbiota; the control group was enriched in three antibiotic biosynthesis pathways, raising the possibility that the healthy lung microbiota possesses the capacity to produce antimicrobial substances that may suppress pathogen overgrowth. The infected group exhibited enrichment of seven pathways related to environmental adaptation, energy metabolism, and signal transduction, suggesting microbial adaptation to the inflamed pulmonary microenvironment [17]. BugBase phenotype prediction further revealed the infected group showed increased potential pathogenicity, Gram-negativity, and stress tolerance, consistent with the enrichment of Proteobacteria (Gram-negative) and depletion of Firmicutes (Gram-positive) in the lung, whereas no significant phenotypic differences were detected in the gut microbiota. Collectively, these predicted features point to a microenvironment that favors opportunistic pathogens and secondary bacterial infections.

Under the experimental conditions of this study, although H9N2 infection did not affect the overall diversity of the gut and lung microbiota, it induced differential enrichment of specific microbial taxa, established a complex network of cross-organ correlations, and drove significant functional and pathogenic phenotype remodeling of the pulmonary microbiota. However, in the future study, quantitative viral load assessment in lung tissue (e.g., RT-qPCR or TCID_50_) should be performed to confirm pulmonary infection and better define its relationship with microbiota changes. In addition, a longitudinal sampling strategy should be employed to distinguish transient from sustained microbiota alterations. These findings reveal the fine-tuning regulatory role of the gut-lung axis in the host response to H9N2 infection.

## Data Availability

The raw sequencing data have been deposited in the NCBI Sequence Read Archive (SRA) under accession number PRJNA1531431.

## Supplementary Materials

The following supporting information can be downloaded at: https://www.mdpi.com/article/10.3390/microorganisms14102219/s1, The complete list of significantly different KEGG pathways for the lung microbiota were provided in Table S1.

## References

[1] Zhang X.H., Zhao Q.Q., Ci X.T., Chen S., Chen L.Y., Lian J.M., Xie Z., Ye Y.Q., Lv H.Y., Li H.X. (2020). Effect of Baicalin on Bacterial Secondary Infection and Inflammation Caused by H9N2 AIV Infection in Chickens. BioMed Res. Int..

[2] Subedi D., Farhan M.H.R., Niraula A., Shrestha P., Chandran D., Acharya K.P., Ahmad M. (2024). Avian Influenza in Low and Middle-Income Countries (LMICs): Outbreaks, Vaccination Challenges and Economic Impact. Pak. Vet. J..

[3] Islam M.T., Roy S., Talukdar H., Shammi S.A., Ahmed S. (2023). Sero-prevalence and associated risk factors of avian influenza virus infection in backyard chicken at Sylhet region, Bangladesh. Int. J. Vet. Sci..

[4] Sagong M., Lee K.N., Lee E.K., Kang H.Y.M., Choi Y.K., Lee Y.J. (2022). Current situation and control strategies of H9N2 avian influenza in South Korea. J. Vet. Sci..

[5] Garaci E., Pariano M., Nunzi E., Costantini C., Bellet M.M., Antognelli C., Russo M.A., Romani L. (2024). Bacteria and fungi of the lung: Allies or enemies?. Front. Pharmacol..

[6] Villena J., Kitazawa H. (2026). The respiratory microbiota: From passive spectator to active guardian of lung immunity. Rev. Argent. Microbiol..

[7] Saint-Martin V., Quéré P., Trapp S., Guabiraba R. (2022). Uncovering the core principles of the gut-lung axis to enhance innate immunity in the chicken. Front. Immunol..

[8] Saint-Martin V., Guillory V., Chollot M., Fleurot I., Kut E., Roesch F., Caballero I., Helloin E., Chambellon E., Ferguson B. (2024). The gut microbiota and its metabolite butyrate shape metabolism and antiviral immunity along the gut-lung axis in the chicken. Commun. Biol..

[9] Sencio V., Machado M.G., Trottein F. (2021). The lung-gut axis during viral respiratory infections: The impact of gut dysbiosis on secondary disease outcomes. Mucosal Immunol..

[10] Davis T., Bialy D., Leng J., La Ragione R., Shelton H., Chrzastek K. (2023). Alteration of the Chicken Upper Respiratory Microbiota, Following H9N2 Avian Influenza Virus Infection. Pathogens.

[11] Abd El-Hack M.E., El-Saadony M.T., Alqhtani A.H., Swelum A.A., Salem H.M., Elbestawy A.R., Noreldin A.E., Babalghith A.O., Khafaga A.F., Hassan M. (2022). The relationship among avian influenza, gut microbiota and chicken immunity: An updated overview. Poult. Sci..

[12] Feng Y., Zhang M., Zhang F., Hou G., Zhang J., Zhao S., Feng Y., Liu D., Xu D., Hu Y. (2026). A microbial consortium constructed with gut microbes of Chinese native chicken breeds protects chicks against *Salmonella* infection. J. Anim. Sci. Biotechnol..

[13] Yitbarek A., Weese J.S., Alkie T.N., Parkinson J., Sharif S. (2018). Influenza A virus subtype H9N2 infection disrupts the composition of intestinal microbiota of chickens. FEMS Microbiol. Ecol..

[14] Reed L.J., Muench H. (1938). A simple method of estimating fifty per cent endpoints. Am. J. Epidemiol..

[15] Chrzastek K., Leng J., Zakaria M.K., Bialy D., La Ragione R., Shelton H. (2021). Low pathogenic avian influenza virus infection retards colon microbiota diversification in two different chicken lines. Anim. Microbiome.

[16] Liu C.S., Zhao D.F., Ma W.J., Guo Y.D., Wang A.J., Wang Q.L., Lee D.J. (2016). Denitrifying sulfide removal process on high-salinity wastewaters in the presence of sp. Appl. Microbiol. Biot..

[17] Oladokun S., Alizadeh M., Mallick A., Fazel F., Doost J.S., Blake K., Denis M.S., Raj S., Sharif S. (2025). Influenza a virus subtype H9N2 infection induces respiratory microbiota dysbiosis in chickens via type-I interferon-mediated mechanisms. FEMS Microbes.

[18] Zhang L., Zhou E.R., Liu C., Tian X.Y., Xue O.D., Zhang K., Luo B. (2024). Avian influenza and gut microbiome in poultry and humans: A “One Health” perspective. Fund. Res..

[19] Berry D., Schwab C., Milinovich G., Reichert J., Ben Mahfoudh K., Decker T., Engel M., Hai B., Hainzl E., Heider S. (2012). Phylotype-level 16S rRNA analysis reveals new bacterial indicators of health state in acute murine colitis. ISME J..

[20] Kenny D.J., Plichta D.R., Shungin D., Koppel N., Hall A.B., Fu B., Vasan R.S., Shaw S.Y., Vlamakis H., Balskus E.P. (2020). Cholesterol Metabolism by Uncultured Human Gut Bacteria Influences Host Cholesterol Level. Cell Host Microbe.

[21] Xing J.H., Niu T.M., Yu T., Zou B.S., Fan S.H., Wang C.F., Shi C.W., Zhang D.X., Wang N., Jiang Y.L. (2025). Gut microbiota-derived isovaleric acid ameliorates influenza virus infection via gut-lung axis. npj Biofilms Microbiomes.

[22] Gonçalves S.M., Lagrou K., Duarte-Oliveira C., Maertens J.A., Cunha C., Carvalho A. (2017). The microbiome-metabolome crosstalk in the pathogenesis of respiratory fungal diseases. Virulence.

[23] Liang X.H., Zhang Z.P., Wang H., Lu X.B., Li W., Lu H.R., Roy A., Shen X.J., Irwin D.M., Shen Y.Y. (2023). Early-life prophylactic antibiotic treatment disturbs the stability of the gut microbiota and increases susceptibility to H9N2 AIV in chicks. Microbiome.

[24] Clark J.A.M., Sun D.M. (2020). Guidelines for the ethical review of laboratory animal welfare People’s Republic of China National Standard GB/T 35892-2018 [Issued 6 February 2018 Effective from 1 September 2018]. Anim. Model Exp. Med..

